# Supplementary material for: Increasing Healthy Food Access for Low-Income Communities: Protocol of the Healthy Community Stores Case Study Project
Source: Int J Environ Res Public Health. 2022 Jan 8;19(2):690. doi: 10.3390/ijerph19020690 (PMC8775718; doi:10.3390/ijerph19020690)
Supplement: Supplementary file 1 [file ijerph-19-00690-s001.zip › Supplementary S1_Interview Guide_Retailers R&R.pdf]

In-depth Interview Guide for Retail Store Owners/Managers (ver 6)

Ice-breaker

1. Tell me about (store name) and the history of the store. What kind of store is (store name)?
    a. Probe: do your customers come to your store for a particular reason/product?
2. How long have you been with [store name]? Tell me about your history with the [store name].
    a. Probe: What is your role at [store name]?
    b. Probe: How long have you been with [store name]?
3. What do you see as the mission or broader goals of (store name)?
4. How do you go about trying to achieve that mission/these goals?

Store Operations Pre-2020 Events

Let's start off by talking about how the store operated BEFORE 2020. We'll get to what happened/is happening in 2020 a bit later.

5. How do you decide on which products to sell in your store? (probe on other reasons than "customer demand")
6. Tell me about how you procure and/or order products for your store.
    a. Probe: who do you work with for sourcing?(Wholesalers/distributors, farmers, other F&V sources)
    b. Probe: describe the logistics of ordering (frequency, delivery, pick-up; etc.)
7. How do you decide which items to procure and stock in your store?
    c. Probe: how important is healthfulness when choosing which products to stock?
    d. Probe: how are decisions made in terms of how much healthy versus unhealthy food to stock?
8. Tell me about how you decide on prices for your products.
    e. Probe: Request examples of how the store goes about pricing of different products
9. Tell me about how you decide where to locate items in the store.
    f. Probe: request examples
10. Tell me about how you decide to promote certain items.
    g. Probe: request examples
    h. Probe: do you have contracts/agreements with companies that require certain product placement and/or in-store promotions?
11. Tell me about your customer base.
    i. Probe: low-income customers, SNAP/WIC participants
12. What kinds of shopping experience do customers have at your store? What do you strive for?
    j. Probe: What do you think customers say about your store?
    k. Probe: What do you think customers like about shopping your store?
    l. Probe: What do you think customers do not like about shopping in your store?

m. Probe: What image or reputation would you like for your store?

Challenges and Adaptations

Now I'm going to ask you think now about the major events of 2020 and the effect it's had on your community and this store.

13. First, tell me about how your region has been impacted (e.g. business closures, public transportation, layoffs, extent of community spread) by the COVID-19 pandemic and other major events such as civil unrest (e.g. protests)?
14. What are your general thoughts on how these events have influenced store operations?
15. Have you noticed that your customer based has changed?
    a. Probe: broader range of customers; more low-income customers?
16. Starting from when the pandemic-related shut downs began, about March 2020, tell me about how you changed things in the store in response?
    b. Probe: Staffing
    c. Probe: Hours
    d. Probe: Store layout
    e. Probe: Safety precautions (PPE, testing)
    f. Probe: Home delivery or other alternative models
    g. Probe: Prices, incentives
    h. Probe: Sourcing/procurement
17. How did your customers react to the changes your store has made?
18. What do you feel were your most successful adaptations during 2020? Why?
    i. Probe: What resources or strategies would you recommend to other retailers in adaptation to the pandemic?
19. Key partners/stakeholders that helped with adaptations this year
20. What were some of the biggest challenges your store has faced during 2020?
21. What challenges do you anticipate for the future?
22. In which areas do you need additional support and resources?
23. Considering the major events of 2020, how do you envision the mission/vision of the store?

<u>Community Engagement and Marketing</u>

24. How does you engage with community members?
    j. Probe: What type of outreach strategies do you use?
    k. Probe: Do you any outreach beyond the local/immediate community?
25. How do you get people in the community to come to your store?

<u>Demographic and Background Questions</u>

- Name of store

- How long have you owned/managed this store?
- Staffing composition
- Acceptance of SNAP/EBT, WIC, or other incentive programs or slide scale
    - % of customers receiving these benefits
    - Barriers or issues in enrolling and operating programs
