# Supplementary material for: Increasing Healthy Food Access for Low-Income Communities: Protocol of the Healthy Community Stores Case Study Project
Source: Int J Environ Res Public Health. 2022 Jan 8;19(2):690. doi: 10.3390/ijerph19020690 (PMC8775718; doi:10.3390/ijerph19020690)
Supplement: Supplementary file 1 [file ijerph-19-00690-s001.zip › Supplementary S2_Interview Guide_Stakeholders_R&R.pdf]

In-depth Interview Guide for Stakeholders (including organizations, companies, community champions/leaders, competing food stores, community residents) (ver 6)

Ice-breaker

1. Tell me about your organization/company and your role there
    a. Probe: What type of services does your organization/company offer?
    b. Probe: What is the mission of your organization/company?
    c. Probe: Who do you serve?

Alternate ice breaker if community champion/resident:

2. Tell me about your community and your role within your community.

General Store Perceptions

3. What are your thoughts about [food retailer]?
4. How do you think your community feels about [food retailer]?

History of Partnership

5. Tell me about your relationship with [food retailer].
    a. Probe: How did that partnership get started? How long have you been working with [food retailers]?
    b. Probe: What has been the scope of your working relationship (e.g. types of projects) with [store retailer]?
    c. Probe: How is this partnership similar/unique to other partnerships your organization has?

If began working with food retailer prior to the beginning 2020, proceed to section, "Stakeholder Engagement with Store: prior to 2020"; if relationship with food retailer began during 2020, skip to section, "Stakeholder Engagement with Store: Changes Related to the Events of 2020"

Stakeholder Engagement with Store: prior to 2020

First, I would like to hear about how you related to (store retailer) before the major events of 2020 (e.g. COVID-19 pandemic, civil unrest)

Success and Challenges

6. What challenges have you faced throughout your work with [food retailer]?
7. What successes have you experienced throughout your work with [food retailer]?
8. Please tell me about a memorable moment you've had with [food retailer]?

    a. (perhaps with staff, the working relationship, a community event, seeing the impact in the community etc.)
9. Provide an example of how you have helped the [food retailer] to improve their store, if applicable?
    a. Probe: "improve" could mean a variety of things such as expand their reach, better serve their customers; etc.
10. Which groups/populations in the community has [food retailer] not successfully connected with yet? Which? How might they do that?
    a. Probe: how well do you think [food retailer] is reaching the low-income population in their community? (Probe for examples)
       Optional: What do you think [food retailer] could do to make its food more appealing to a larger number of people?

<u>Stakeholder Engagement with Store: Changes Related to the Events of 2020</u>

Now we would like to hear about how you have adapted your work in response to the major events of 2020 (e.g. COVID-19 pandemic, civil unrest).

11. What do you consider to be the most impactful event(s) in your region during 2020? Please describe.

Adaptations: Organization

12. How have you/your organization/company changed its practices in response to the major events of 2020?

Adaptations: Partnership with [food retailer]

13. Tell me about the ways in which you/your organization partnered with [food retailer] as they adapted throughout 2020.
    a. Probe: *If began working together at the onset of the pandemic:* How did you/your organization get involved?
    b. Probe: *If began working together prior to the onset of the pandemic:* How did you/your partnership with [food retailer] change throughout 2020?

Success and Challenges

14. What challenges have you faced throughout your work with [food retailer] in 2020?
15. What successes have you experienced throughout your work with [food retailer] in 2020?
16. Please tell me about a memorable moment you've had with [food retailer] in 2020?
    a. (perhaps with staff, the working relationship, a community event, seeing the impact in the community etc.)
17. Provide an example of how you have helped the [food retailer] to improve their store in 2020?

    a. Probe: "improve" could mean a variety of things such as expand their reach, better serve their customers; etc.

18. Which groups/populations in the community has [food retailer] not successfully connected with yet in 2020? Which? How might they do that?
    a. Probe: how well do you think [food retailer] is reaching the low-income population in their community?
    b. Optional: What do you think [food retailer] could do to make its food more appealing to a larger number of people?

Suggestions

19. If the retailer were to ask for your suggestions on how to improve, what would you say?
    a. Probe: "improve" could mean a variety of things such as expand their reach, better serve their customers
20. Who are the other stakeholders, including funders, that are crucial to your organization's success?

Future Directions

21. What do you envision for the future of your partnership with [food retailer}?

<u>Demographic and Background Questions</u>

22. How many years have you been working at [organization]?
23. What type of experienced, training and/or education prepared you for your current role?
24. How would you describe your relationship with the communities served by your organization? *[Note to interviewer: do not read answers, but may confirm which they fall into]*
    a. *I consider myself a member of the target communities (select one or more of the below answers)*
        a. *Lives in the same neighborhood*
        b. *Similar economic background*
        c. *Same race or ethnicity*
    b. *I am not a member of the target communities, but I have been working with them for a long time and am trusted by community members*
    c. *I do not consider myself a member of the target community*

<u>Closing Questions</u>

- Is there anything else you would like to share with us?
- Is there anyone else that you think we should talk to?
