# Supplementary material for: Increasing Healthy Food Access for Low-Income Communities: Protocol of the Healthy Community Stores Case Study Project
Source: Int J Environ Res Public Health. 2022 Jan 8;19(2):690. doi: 10.3390/ijerph19020690 (PMC8775718; doi:10.3390/ijerph19020690)
Supplement: Supplementary file 1 [file ijerph-19-00690-s001.zip › Supplementary S3_Case Study Report Outline_R&R.pdf]

<u>Case Study Report Overview</u>

General Format:

A. Single spaced, plus appendices
B. Relevant quotations embedded throughout (indented if >1 sentence)
C. Integration of data sources (qual and quant) within each section (also including lay press articles, photos
D. Use of tables/figures in common formats → to be discussed

Report Outline

1) Executive Summary

2) Introduction
   a) Store Setting
      i) Physical, social, cultural, historical context of the store
         (1) Organizational details (owner/manager background, staff composition)
         (2) Description of surrounding community
      ii) Economic context (e.g., business model, funding sources)
   b) Mission of the store (e.g., health focus)

3) Store Operations (prior to the events of 2020)
   a) Food stocking and sales
      i) Decision-making process
      ii) Sourcing strategies
      iii) Pricing strategies
   b) Community engagement
      i) Customer demographic
      ii) Customer shopping experience
      iii) Outreach/marketing strategies

4) Regional impact of events of 2020
   a) Description of the events that have impacted the store's region
   b) How these events affect the store

5) Store operations (2020 – present) *attention to patterns and practices that differ from prior to 2020*
   a) Overview of operational changes that occurred in store (e.g., staffing, hours, layout, safety, home delivery; etc.)
   b) Overview of changes in sourcing/procurement and pricing
      i) Includes findings from adapted NEMS-S assessment and sales assessment tool

c) Overview of changes with community engagement
    i) Customer demographic
    ii) Customer shopping experience
    iii) Outreach/marketing strategies
d) Key partners/stakeholders that supported the store's adaptations and their involvement *[need to decide if this can be integrated into section E. or should have a separate section]*

6) Lessons learned
    a) Successes – key adaptations that were successful
    b) Challenges – adaptations that did not go well; areas in which they need additional support and resources

7) Future Directions
    a) Outlook for the food retailer: struggling/expanding?
        i) Anticipated challenges
    b) Future plans (e.g., going online, etc.)

8) Implications
    a) Retailer implications: how can these findings be used to inform the practices of similar food retailers looking to adapt in this setting or others?
    b) Policy implications: what would keep this store from functioning to its full extent?
